# Antenatal HIV-1 RNA load and timing of mother to child transmission; a nested case-control study in a resource poor setting

**DOI:** 10.1186/1743-422X-7-176

**Published:** 2010-08-02

**Authors:** Kerina Duri, Felicity Z Gumbo, Knut I Kristiansen, Nyaradzi E Kurewa, Munyaradzi P Mapingure, Simbarashe Rusakaniko, Mike Z Chirenje, Fredrik Muller, Babill Stray-Pedersen

**Affiliations:** 1 Department of Immunology, University of Zimbabwe, Harare, Zimbabwe; 2 Department of Paediatrics and Child Health, University of Zimbabwe, Harare, Zimbabwe; 3 Institute of Microbiology, University of Oslo and Rikshospitalet, Oslo University Hospital, Oslo, Norway; 4 Division of Obstetrics and Gynecology, University of Oslo, Oslo, Norway; 5 Department of Community Medicine, University of Zimbabwe, Harare, Zimbabwe; 6 Department of Obstetrics and Gynecology, University of Zimbabwe, Harare, Zimbabwe

## Abstract

**Objective:**

To determine HIV-1 RNA load during the third trimester of pregnancy and evaluate its effect on *in utero *and intra-partum/postpartum transmissions in a breastfeeding population.

**Design:**

A nested case-control study within a PMTCT cohort of antiretroviral therapy naive pregnant women and their infants.

**Methods:**

A case was a mother who transmitted HIV-1 to her infant (transmitter) who was matched to one HIV-1 positive but non-transmitting mother (control).

**Results:**

From a cohort of 691 pregnant women, 177 (25.6%) were HIV-1 positive at enrolment and from these 29 (23%) transmitted HIV-1 to their infants, 10 and 19 during *in utero *and intra-partum/postpartum respectively. Twenty-four mothers sero-converted after delivery and three transmitted HIV-1 to their infants. Each unit increase in log_10 _viral load was associated with a 178 cells/mm^3 ^and 0.2 g/dL decrease in TLC and hemoglobin levels, p = 0.048 and 0.021 respectively, and a 29% increase in the risk of transmission, p = 0.023. Intra-partum/postpartum transmitters had significantly higher mean viral load relative to their matched controls, p = 0.034.

**Conclusion:**

Antenatal serum HIV-1 RNA load, TLC and hemoglobin levels were significantly associated with vertical transmission but this association was independent of transmission time. This finding supports the rationale for preventive strategies designed to reduce vertical transmission by lowering maternal viral load.

## Introduction

Sub-Saharan Africa continues to be the epicentre of the HIV-1 epidemic contributing more than 90% of the 370 000 infants who acquire the infection from their mothers annually worldwide [1]. More than half of the HIV-1 infected children die before their second birthday [2]. The HIV-1 epidemic among pregnant women poses a challenge to child health and survival of future generations.

Zimbabwe is among the Sub-Saharan countries with the highest HIV-1 prevalence in the world. Among 600 000 women who get pregnant annually, HIV-1 prevalence peaked to 30% in 1997 [3] but has steadily declined over the years to 15.6% in 2006 [1,4]. Without any intervention, 30-49% of the children born to HIV-1 positive mothers are infected by the virus [5]. In Zimbabwe the estimate of mother to child transmission rate of HIV-1 has been shown to be 30% [6]. The reason why some mothers transmit to their infants whilst the majority does not is not well documented.

Maternal HIV-1 RNA load has been shown to be the strongest predictor of vertical transmission [7,8]. In Zimbabwe, among exclusively breastfed infants, *in utero *and intra-partum transmission has been shown to be 9.4% and 16%, respectively [6] with a postpartum transmission rate of 12% [9]. However, both studies have made no reference to maternal viral load. More so, other previous studies have pooled the three transmission periods; *in utero*, intra-partum and postpartum cases and this may underestimate time specific risk factors of vertical transmission [8].

Despite the high HIV-1 prevalence in the general populace which translates to high vertical transmission rates, the desire to have future pregnancies among HIV-1 positive mothers has increased from 3% to more than 55% more so with the advent of HIV-1 Prevention of Mother To Child Transmission (PMTCT) initiatives [10,11]. Therefore there is a need for the development of a simple, effective and time specific vertical transmission preventive strategy to curb this epidemic. This study aims to determine HIV-1 RNA load during the third trimester of pregnancy and evaluate its association with *in utero *and intra-partum/postpartum vertical transmissions.

## Methodology

### Study Design and Setting

This was a nested case-control study in which the cases and controls were sampled from an antiretroviral therapy naive PMTCT cohort of pregnant women attending Antenatal Clinics at Epworth, Seke North and Saint Mary's Chitungwiza, all around Harare. Antiretroviral drugs were not readily available in Zimbabwe at the time of recruitment of study participants.

### Study Population and Procedures

The study population consisted of two groups of HIV-1 positive pregnant women. The main group comprised of pregnant women who were HIV-1 positive at enrolment, referred to as having chronic HIV-1 infections and a minor group of women who were HIV-1 negative during pregnancy but later on sero-converted after delivery during the follow-up period, regarded as having acute HIV-1 infections. Each HIV-1 positive mother who transmitted the virus to her infant (case) was matched to one HIV-1 positive but non-transmitting mother (control). Matching of cases and controls was done with respect to maternal age, educational level, marital and socio-economic status, parity, alcohol intake, sexually transmitted infections, the date of last menstruation, and uptake of single dose nevirapine therapy.

Pregnant women were enrolled at 36 gestational weeks in a national PMTCT program between April and September 2002. Pre-and post-HIV test counseling was provided. Single dose nevirapine therapy was offered to HIV-1 positive mothers during labour and to their infants within 72 hours post delivery. Mothers were encouraged to exclusively breastfeed during the first six months. Follow-up was from delivery, six weeks, four and nine months and thereafter three monthly until two years. Follow up visits generally coincided with infant immunization visits. At each subsequent follow-up visit, HIV-1 negative mother and infants were re-tested for HIV-1 antibodies and HIV-1 DNA, respectively. Serum samples from the HIV-1 negative mothers and their infants were aliquoted and appropriately stored for further tests in the event that they sero-converted.

### Mothers and Infants Demographic characteristics and Examination

All mothers answered a structured questionnaire at enrolment and information regarding their socio-demographics, sexual behavior, obstetric and reproductive health issues was obtained. A gynecologist performed physical and gynecological examinations.

A pediatrician examined infants. Date of birth, birth weight, gender, single dose nevirapine therapy and breastfeeding patterns were recorded. Infant deaths were also recorded during the follow up period.

### Mothers' Tests

Serial HIV-1/2 algorithm antibody tests were done using Determine (Abbott Diagnostics, Illinois USA) and Ora-Quick (Abbott Diagnostics, Illinois, USA) rapid kits on mothers' serum samples. EDTA-anti-coagulated venous blood samples were processed within six hours for full blood counts using Abbott Diagnostic Cell Dyne 3500R SL Hematology Analyser. Total Lymphocyte Count (TLC) was enumerated as the total white blood cell count multiplied by the lymphocyte percentage. In this resource poor setting, TLC was used as a surrogate marker for CD4 cell count since by then, the capacity to determine the latter was not readily available to the general public due to prohibitive costs. TLC of 1200 cells/mm^3 ^was the threshold value used equivalent to a CD4 count of 200 cells/mm^3 ^[12,13].

Blood samples were shipped on dry ice to the Institute of Microbiology at the University of Oslo in Norway for further laboratory analysis. Maternal baseline serum samples were quantified for HIV-1 RNA load using an automated TaqMan Roche Amplicor 1.5 Monitor Test (Cobas AmpliPrep/Cobas TaqMan, Roche Diagnostics, Branchburg NJ) according to the manufacturer's instructions. As for sero-converters, the first HIV-1 positive sample was quantified. The linear range of the test was between 40 (1.6log_10_) and 10^7 ^(7log_10_) copies/mL and the detection limit of the assay is 40 copies/mL based on a sample volume of 1 mL thus the detection limit for this study was 400 copies/mL based on a serum sample volume of 100 μL that was topped up to 1 mL with HIV-1 negative serum.

### Infants' Tests

Infants' venous EDTA whole blood samples were collected at each follow up visit. Samples were stored at -86°C until testing. Detection of infants' HIV-1 infection was determined using a qualitative 1.5 Roche Amplicor HIV-1 DNA PCR kit (Roche Diagnostics Incorporation, Branchburg, New Jersey). Testing was done in the Obstetrics and Gynecology Department, Medical School, University of Zimbabwe. Infants that tested HIV-1 DNA PCR positive on whole blood collected within 10 days of birth were considered to be infected *in utero*. Infants who had negative HIV-1 DNA PCR results within the first 10 days of life and positive results at six weeks postpartum and/or thereafter were considered to be infected intra-partum/postpartum [14].

### Statistical Analysis

Data were collected and analyzed using STATA version 10 from Texas and SPSS version 16.0 from Illinois, USA. Viral load values were log_10 _transformed. Viral load values of below the detection limit were assigned half the value of the detection limit. The Student t-test was used to compare mean log_10 _viral load between transmitting and non transmitting mothers, chronic and acute HIV-1 infections, *in utero *and intra-partum/postpartum transmitters. Mean log_10 _viral load of each of these groups was also compared with their respective matched controls. Regression analysis was used to investigate the association between log_10 _viral load, TLC or hemoglobin levels, and vertical transmission. Tests of statistical significance included the 95% confidence interval of relative risks, two sided p values based on Chi-squared and Fisher's exact tests.

### Ethical Consideration

The study was approved by the Medical Research Council of Zimbabwe and the Ethical Review Committee in Norway. Written consent to participate in the research study was obtained from the mothers and they were free to discontinue at any given time without any prejudice. Mothers also consented to have their blood samples and that of their index infants' used for future HIV related research.

## Results

### Demographic and reproductive health characteristics of 32 transmitters and matched 32 non-transmitters

There was no statistical significant difference with respect to socio-demographic characteristics, sexual behavior, reproductive genital tract infections and medical history between the 64 HIV-1 positive mothers constituting this study population and the rest (113) of the HIV-1 positive mothers in the cohort. However, the 32 transmitters and matched 32 non-transmitters were more likely to have more children relative to the other 113 HIV-1 positive but non transmitting mothers who were not part of the study population, p = 0.016.

Mothers' mean age (SD) was 26.0 (5.6) years with that of transmitters and non-transmitters being 26.3 (5.6) and 25.6 (5.6) years respectively p = 0.610. All the mothers had spontaneous vaginal deliveries. There were no statistically significant differences between transmitting and non-transmitting mothers with respect to age, level of education, parity, type of marriage, socio-economic status and number of life sexual partners. The transmitters and non-transmitters also had comparable burdens of reproductive tract infections and obstetric history, see table 1.

**Table 1:** Socio-demographics, sexual behavior, medical history and baby characteristics of the 32 transmitters and 32 non-transmitters

Variable	Transmitter N = 32 (%)	Non Transmitters N = 32 (%)	RR (95% CI)
Age in years			
Mean (sd)	26.3 (5.6)	25.6 (5.6)	1.01 (0.95-1.08)
Years in school			
< 8	4/32 (13)	3/32 (9)	1.16 (0.58-2.33)
Parity			
At least 1 child	28/32 (88)	25/32 (78)	1.40 (0.60-3.30)
Polygamous marriage			
Yes	4/31 (13)	4/30 (13)	0.98 (0.47-2.06)
Subsidised income			
Yes	4/32 (13)	8/32 (25)	0.62 (0.27-1.43)
Age at sexual debut			
≤ 16 years	6/32 (19)	3/32 (9)	1.41 (0.82-2.42)
Life time partners			
> 1	17/32 (53)	16/32 (50)	1.06 (0.65-1.74)
Vaginal discharge			
Abnormal	15/32 (47)	13/31 (42)	1.10 (0.68-1.79)
Genital ulcer			
Present	5/32 (16)	3/30 (10)	1.25 (0.69-2.28)
Dysuria			
Yes	7/32 (22)	4/31 (13)	1.32 (0.78-2.25)
Lymphadenopathy			
Yes	3/28 (11)	1/31 (3)	1.65 (0.87-3.12)
Abortion history			
Yes	7/32 (22)	3/32 (9)	1.51 (0.92-2.49)
Infant death history			
Yes	4/32 (13)	7/32 (22)	0.69 (0.30-1.57)
Schistosomiasis infection history			
Yes	7/32 (22)	4/31 (13)	1.32 (0.78-2.24)
Mothers'ARV Prophylaxis			
No	17/32 (53)	16/32 (50)	1.00 (0.60-1.66)
Infant gender			
Male	14/30 (47)	12/29 (41)	1.11 (0.67-1.83)
Birth weight			
<2500	2/31 (6)	1/30 (3)	1.36 (0.58-3.15)
Deceased infant			
Yes	9/31 (29)	2/30 (7)	4.35 (1.02-18.52)*
Breastfed			
Yes	28/31 (90)	19/25 (76)	1.79 (0.69-4.64)
Baby ARV prophylaxis			
Yes	16/27 (59)	13/25 (52)	0.87 (0.51-1.49)

When the transmitters were stratified by time of infecting their infants, there were no statistically significant differences with respect to demographics, sexual behaviour, reproductive health characteristics and medical history between those who transmitted during *in utero *and those who transmitted intra-partum/postpartum.

Mothers with acute HIV-1 infection, the sero-converters, were generally younger relative to HIV-1 negative mothers in the cohort, with mean age of 21.8 (4.6) and 23.7(5) years respectively although not statistically significant, p = 0.06. There were also no statistical significant differences with respect to parity, level of education, age of sexual debut and reproductive tract infections between these two groups. Sero-converters were more likely to be single, have more than one sexual partner(s), syphilis, clinical warts, and a history of blood transfusion with p values of 0.000, 0.019, 0.041, 0.002 and 0.033 respectively. Transmitting sero-converters were more likely to report having a travelling partner, p = 0.022 and were significantly younger than transmitters with chronic HIV-1 infections, with mean age of 20(1.7) and 27(5.5) years respectively, p = 0.04.

### HIV-1 Prevalence and Transmission

**Figure 1:**
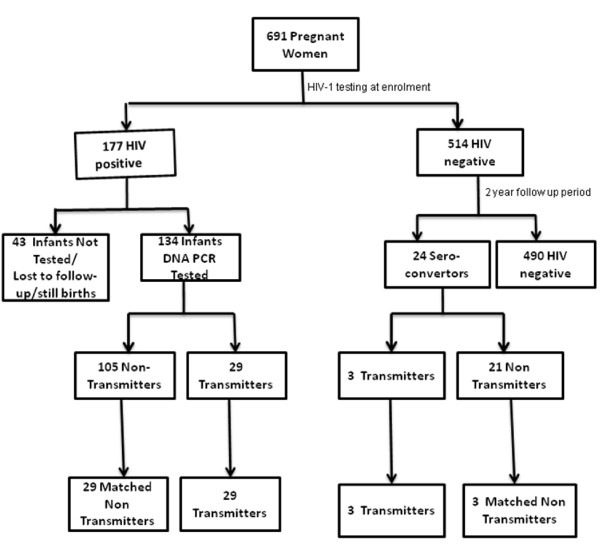
**Summary of the sampling of the 32 transmitters and 32 non-transmitters**.

At enrolment, 691 pregnant women attending national PMTCT program were sampled between April and September 2002. Of these, 177 (25.6%) and 514 (74.4%) were HIV-1 positive and negative respectively. There were two stillbirths each among the HIV-1 positive and negative mothers and these were excluded from analysis. From the 176 mothers with chronic HIV-1 infections that delivered live births 134 (76%) mother-baby pairs were successfully followed up and tested, see figure 1. There were no statistically significant differences with respect to socio-demography and reproductive health characteristics between the 42 women lost to follow up and the 134 with complete data sets. Twenty-nine (22%) mothers transmitted the virus to their infants, 10 (34%) and 19 (66%) during *in utero *and intra-partum/postpartum transmissions with rates of 7.5% and 15.3% respectively.

Out of the 514 HIV-1 negative mothers at baseline, 24 sero-converted during the 2 year follow-up period, giving an HIV-1 cumulative incidence rate of 2.3 per hundred women years. Among the 24 sero-converters with acute HIV-1 infections, three (13%) transmitted the virus to their infants through breastfeeding around 9 months postpartum. All the three infants were exposed, through breast milk for about three months before acquiring HIV-1 infection at about 12 months postpartum.

Thus there were a total of 32 transmitting mothers in this cohort, giving an overall transmission rate of 21.3%.

### Maternal Viral Load and Transmission

Of the 32 transmitters and 32 matched non-transmitters, 26 (81%) and 20 (63%), respectively had detectable serum HIV-1 RNA load ranging from 400 to 3 000 000 copies/mL. Vertical transmission occurred throughout the entire range with 90% of the transmissions occurring below 16 000 HIV-1 RNA copies/mL. The mean (95% Confidence Interval) log_10 _viral load was 3.55(3.15-3.96) and 2.92(2.59-3.26) for transmitters and non-transmitters respectively, p = 0.018, see table 2. For each unit increase in log_10 _viral load, the risk of transmission increased by 29%, p = 0.023. Mean log_10 _(SD) viral load of mothers with acute and chronic HIV-1 infection was 4.22 (1.01) and 3.55 (1.09) respectively, p = 0.317. Mean log_10 _(SD) viral load of transmitting sero-converters and non-transmitting sero-converters were 3.99 (1.34) and 2.77 (0.81) respectively, p = 0.248. There was no statistical significant difference in mean log_10 _viral load between *in utero *and intra-partum/postpartum transmitters. *In-utero *transmitters generally had higher mean log_10 _viral load compared to their matched controls though not statistically significant and similarly intra-partum/postpartum transmitters had significantly higher mean viral load relative to their respective matched controls, p = 0.034.

**Table 2:** Baseline HIV-1 RNA load, TLC and hemoglobin levels of 32 transmitters and non-transmitters

Variable	Transmitters N = 32	Non-transmitters N = 32	RR (95% CI)
Hemoglobin g/dl			
<10	7/32 (22)	2/32 (6)	1.71 (1.08-2.69)*
TLC			
Mean cells/mm^3^(sd)	2147 (111)	2505 (132)	0.99 (0.99-0.99)*
Viral load			
Mean log10 copies/ml	3.55 (1.12)	2.92 (0.92)	1.29 (1.07-1.55)*

Among the 32 transmitting and 32 non-transmitting mothers 6 (19%) and 12 (37.5%), respectively had undetectable viral load respectively and none of them were from the acute infection subgroup. Mothers with undetectable viral load were less likely to transmit when compared to mothers with detectable viral load. There were no statistically significant differences regarding socio-demographic and reproductive health characteristics between mothers with detectable and undetectable viral loads.

### HIV-1 RNA load, TLC, Hemoglobin levels and Transmission

Mean TLC for transmitting mothers and non-transmitting mothers were 2147 cells/mm^3 ^and 2505 cells/mm^3 ^respectively, p = 0.04. HIV-1 RNA load negatively correlated with TLC, correlation coefficient of -0.254. Each unit increase in log_10 _viral load was associated with a 178 cells/mm^3 ^decrease in TLC, (p = 0.048). There were no statistical significant differences in mean TLC of mothers with acute and chronic HIV-1 infections and also between *in utero *transmitters and their respective controls. However, there was a statistically significant difference in mean TLC between intra-partum/postpartum transmitters and their matched controls, p = 0.030, see table 3.

**Table 3:** Comparison of *in utero *and intra-partum/postpartum transmitters and their respective matched non-transmitting controls with respect to viral load, TLC and hemoglobin levels

Variable	*In utero *transmitters N = 10	In utero matched controls N = 10	RR (95%CI)	Intra/Postpartum Transmitters N = 22	Intra-/postpartum Matched Controls N = 22	RR (95% CI)
Hemoglobin						
Mean g/dl (sd)	10.7 (0.9)	10.5 (1.3)	1.10 (0.74-1.65)	10.5 (1.5)	11.4 (1.0)	0.82 (0.71-0.94)*
TLC						
Mean cells/ml (SD)	2133 (724)	2207 (443)	0.99 (0.98-1.00)	2153 (576)	2632 (790)	0.98 (0.97 = 0.99)*
Viral load						
Mean log10 copies/ml	3.5 (1.2)	3.0 (0.9)	1.60 (0.70-3.79)	3.6 (1.1)	2.9 (1.0)	2.0 (1.02-3.81)*

Each unit increase in log_10 _viral load was associated with a 0.2 g/dL decrease in hemoglobin levels, p = 0.021. There were no statistically significant differences in hemoglobin levels between mothers with acute and chronic HIV-1 infection and also among *in utero *and intra-partum/postpartum transmitters, p = 0.870 and 0.980 respectively. Mean hemoglobin levels were significantly different between intra-partum/postpartum transmitters relative to their matched controls p = 0.038, see table 3. Anaemic mothers with hemoglobin levels of less than 10 g/dL were 1.7 times more likely to transmit compared to those with hemoglobin levels of more than 10 g/dL in univariate analysis. After controlling for the effect of viral load and TLC this relationship ceased to be significant.

### Infant Factors, Mortality and Transmission

Infant sex, birth weight, single dose nevirapine therapy and breastfeeding patterns were not significantly different neither between transmitters and non-transmitters nor among *in utero *and intra-partum/postpartum transmitters. HIV-1 infected infants were 4 times more likely to die compared to those uninfected (p = 0.003), see table 1. The odds of dying were 14 (p = 0.04) for infants infected *in utero *compared to their respective uninfected controls.

## Discussion

This is a first study in Zimbabwe where viral load was determined in pregnant women and was related to time point vertical transmission. This nested case-control study of Harare peri-urban pregnant women provided data on risk factors of vertical transmission by assessing maternal HIV-1 RNA load, TLC and hemoglobin levels of transmitting and non-transmitting mothers, who were otherwise similar with respect to demographic and reproductive health characteristics.

Of note was the highly significant relationship between antenatal HIV-1 RNA load, at 36 weeks gestational period, with vertical transmission. Similar to other studies [7,8,15,16], transmitting mothers had a significantly higher viral load compared to non-transmitting mothers.

No threshold for transmission was observed in this cohort that could predict transmission or non-transmission, as transmission occurred throughout the whole range of viral load values, contrary to previous studies [17]. More so, no threshold of HIV-1 RNA load was associated with *in utero *and intra-partum/postpartum transmissions contrary to some studies [18-20]. Our findings are analogous to those by Garcia *et al*., where serum HIV-1 RNA levels predicted the risk but not the timing of vertical transmission [21]. While viral load was an important determinant of vertical transmission, it was not the only one, as six percent of non-transmitting mothers had high viral loads of >100 000 copies/mL yet they did not transmit. Besides high levels of viremia, other risk factors of vertical transmission such as maternal host genetic factors, neutralizing antibodies, HIV-1 phenotype and/or genetic diversity could have also played a role in transmission.

Eighteen (28%) of the 64 mothers had undetectable viral load yet some (n = 6) still transmitted the virus to their infants. A Spanish study has also observed some pregnant women with undetectable plasma viral load who were at risk of vertically transmitting the HIV-1 RNA during vaginal delivery [22]. Quantification of HIV-1 RNA in cervico-vaginal secretions has been shown to be more useful when investigating vertical transmission risk associated with vaginal delivery [23]. African mothers who are immigrants in Europe have been shown to have lower HIV-1 RNA loads but were more likely to vertically transmit relative to their non-African counterparts [23-26] probably due to differences in HIV-1 subtypes and host genetic factors. This group of mothers with undetectable viral load could be elite controllers [27-29]. Elite controllers have been shown to maintain high levels of CD4^+ ^CD25^+ ^regulatory T cells in their peripheral blood [30]. These are of high research interest as they may provide novel insights regarding host mechanism of virus control. The percentage of the mothers with undetectable viral load in this study was relatively higher compared to previous Zimbabwean studies done in the late 1990 s which was around 10% [8,31]. This could be attributed to differences in quantitation methods used. The fully automated COBAS AmpliPrep/COBAS TaqMan Viral RNA load test used has been shown to excellently satisfy the requirements for reliable quantification of HIV-1 RNA in clinical specimens of all HIV-1 subtypes [32,33] and the automation itself reduced inter and intra assay variation.

Infant HIV-1 status was successfully determined using qualitative Roche DNA PCR. This test has shown 100% sensitivity and 100% specificity at least in Zimbabwean infants and adults with predominant HIV-1 subtype C [6,34]. The observed *in utero *and intra-partum/postpartum transmission rates of 7.5%, and 15.3% were quite comparable but lower relative to a previous Zimbabwean study that has shown *in utero*, intra-partum/early postpartum and late postpartum transmission rates of 9.4%, 16% and 5.3%, respectively [6]. The rates were also quite comparable to those obtained in a Tanzanian cohort with an *in utero *and intra-partum transmission rates of 8.4% and 16.1% respectively [19]. The overall vertical transmission rate of 21.3% observed was much lower compared to that obtained from previous studies prior to antiretroviral prophylaxis era of 30.7% and 27% [6,8]. This coincides with the general decrease in HIV-1 prevalence in the general population and could be attributed to better access to antiretroviral prophylaxis. However, in this cohort receiving single dose nevirapine was not protective against HIV-1 vertical transmission [35]. This could possibly be due to a relatively small sample size. Intra-partum/postpartum transmissions constituted the majority, 69% of the infections. Other African studies have also shown such high transmission rates through breastfeeding [36]. In resource poor settings, where a large proportion of infants are infected through breastfeeding, concerted efforts should be made towards interventions aimed at reducing such transmissions by advocating for more effective HAART during pregnancy and or breastfeeding, encouraging exclusive breastfeeding for six months, with ongoing breastfeeding thereafter, during the introductions of complementary feeds [37].

Generally male infants were more at risk of HIV-1 vertical transmission though this was not statistically significant, unlike previous studies where *in utero *transmission was significantly higher among girl than boy infants [38]. Consistent with other studies was the fact that, *in utero *infected infants were 2.5 times more likely to die relative to intra-partum infected infants probably because they would have been infected for longer periods [39].

As early as 1964, it was recognized that a decrease in the TLC was associated with immune suppression [40]. The equipment and skills to perform total white blood cell count and differentials are readily available in most hospitals and clinics in resource-poor settings, and performing a TLC costs much cheaper compared to CD4 cell count measurements. We applied WHO guidelines that acknowledge that TLC may be used as surrogate marker for CD4 counts in situations where CD4 cell count measurements may not be affordable. Observed was a negative correlation between HIV-1 RNA load and TLC. Pregnant women with high TLC were less likely to transmit to their infants compared to those with low counts and such findings have been observed by others [16,18]. Anaemic mothers were more likely to transmit to their babies. A mean decline of 0.46 g/dL hemoglobin level per unit increase in log_10 _viral load has been observed in a South African study [41]. This value is relatively higher compared to 0.2 g/dL decrease in hemoglobin levels observed in our study. This is probably due to the fact that the former study sampled only patients with acute HIV-1 infection which was not the case with our study.

In this cohort being single, having multiple partners and having a history of blood transfusion constituted significant risk factors for HIV-1 sero-conversion following delivery. Transmitting sero converters were more likely to be young and have a travelling partner. Prevention strategies should address these risk factors associated with sero-conversion to reduce HIV-1 incidence rates in the general population. In such poor resource settings a nested case control design reduced costs and efforts of data collection considerably with relatively minor loss in statistical efficiency [42]. However, all transmitting and non transmitting mothers selected in the study may not be a full representation of all the cases and controls in the original cohort due to failure to follow up all the mothers and infants, though generally the follow-up rate was good.

## Conclusion

We concluded that antenatal serum HIV-1 RNA viral load, TLC and hemoglobin levels in the third trimester were significantly associated with vertical transmission and this association was independent of transmission time. These data support the rationale for preventive strategies designed to reduce vertical transmission through lowering maternal viral load by introducing more effective HAART during pregnancy, delivery and breastfeeding. Unclear are the factors that contribute to the low viral load levels which were observed in some transmitting mothers. Further research is warranted to determine host genetic factors among these mothers who had undetectable viral load but still transmitted to their infants.

## Competing interests

The authors declare that they have no competing interests.

## Authors' contributions

DK collected data, carried out the laboratory analysis and drafted the manuscript, GFZ collected data, KKI participated in laboratory analysis, KNE collected data, MMP carried out data analysis and interpretation of results, RS supervised data analysis and interpretation of results, CMZ participated in designing of the study, MF supervised laboratory analysis, SB participated in designing of the study. All authors read and corrected the final version of the manuscript.
